# Heart Failure with Preserved Left Ventricular Ejection Fraction in Patients with Acute Myocardial Infarction

**DOI:** 10.5935/abc.20150055

**Published:** 2015-08

**Authors:** Lucas Antonelli, Marcelo Katz, Fernando Bacal, Marcia Regina Pinho Makdisse, Alessandra Graça Correa, Carolina Pereira, Marcelo Franken, Anderson Nunes Fava, Carlos Vicente Serrano Junior, Antonio Eduardo Pereira Pesaro

**Affiliations:** Hospital Israelita Albert Einstein (HIAE), São Paulo, SP – Brazil

**Keywords:** Heart Failure, Myocardial Infarction, Stroke Volume, Prevalence

## Abstract

**Background:**

The prevalence and clinical outcomes of heart failure with preserved left ventricular ejection fraction after acute myocardial infarction have not been well elucidated.

**Objective:**

To analyze the prevalence of heart failure with preserved left ventricular ejection fraction in acute myocardial infarction and its association with mortality.

**Methods:**

Patients with acute myocardial infarction (n = 1,474) were prospectively included. Patients without heart failure (Killip score = 1), with heart failure with preserved left ventricular ejection fraction (Killip score > 1 and left ventricle ejection fraction ≥ 50%), and with systolic dysfunction (Killip score > 1 and left ventricle ejection fraction < 50%) on admission were compared. The association between systolic dysfunction with preserved left ventricular ejection fraction and in-hospital mortality was tested in adjusted models.

**Results:**

Among the patients included, 1,256 (85.2%) were admitted without heart failure (72% men, 67 ± 15 years), 78 (5.3%) with heart failure with preserved left ventricular ejection fraction (59% men, 76 ± 14 years), and 140 (9.5%) with systolic dysfunction (69% men, 76 ± 14 years), with mortality rates of 4.3%, 17.9%, and 27.1%, respectively (p < 0.001). Logistic regression (adjusted for sex, age, troponin, diabetes, and body mass index) demonstrated that heart failure with preserved left ventricular ejection fraction (OR 2.91; 95% CI 1.35–6.27; p = 0.006) and systolic dysfunction (OR 5.38; 95% CI 3.10 to 9.32; p < 0.001) were associated with in-hospital mortality.

**Conclusion:**

One-third of patients with acute myocardial infarction admitted with heart failure had preserved left ventricular ejection fraction. Although this subgroup exhibited more favorable outcomes than those with systolic dysfunction, this condition presented a three-fold higher risk of death than the group without heart failure. Patients with acute myocardial infarction and heart failure with preserved left ventricular ejection fraction encounter elevated short-term risk and require special attention and monitoring during hospitalization.

## Introduction

Diastolic heart failure (HF) is a clinical syndrome defined by the presence of signs and symptoms of HF, preserved left ventricle ejection fraction (LVEF), and abnormal diastolic function^1^. It is characterized by an abnormality in ventricular distensibility, relaxation, and filling, all of which can be indirectly measured by echocardiography^2^. In the absence of echocardiographic assessment of diastolic function, HF with LVEF ≥ 50% can be termed only “HF with preserved LVEF.” Although patients with HF with preserved LVEF generally present a more favorable prognosis than those with systolic dysfunction, there is increasing morbidity related to HF with preserved LVEF due to population aging and therapeutic limitations associated with this pathology.

In particular, systolic dysfunction is an important marker of poor prognosis in acute myocardial infarction (AMI)^3,4^. Conversely, the presence of diastolic dysfunction, whether associated with systolic dysfunction, is an aggravating factor that is associated with poor prognosis in this situation^5,6^. Previous studies have suggested that the development of HF after AMI is related to the infarction size, coronary multivessel disease, reperfusion efficiency, and adjuvant medication use^7-9^. Despite the increasing use of early myocardial revascularization^8^, the prevalence of post-AMI HF is still high (20%–30%), representing the leading cause of in-hospital mortality^6,10^. Systolic ventricular dysfunction after AMI in relation to the development of HF and increased mortality has been extensively studied. Moreover, data relating to the prevalence and prognosis of patients with post-AMI HF with preserved LVEF are still limited^11^. A few registries have specifically evaluated post-AMI HF with preserved LVEF; however, they generally have not simultaneously assessed AMI patients with and without ST-segment elevation (STEMI and NSTEMI, respectively) and have used heterogenous LVEF cut-off points to establish the diagnosis of HF with preserved LVEF^12-14^. Here we aim to evaluate the prevalence, clinical characteristics, and clinical outcomes of patients admitted with post-AMI HF with preserved LVEF.

## Methods

Between January 2005 and December 2012, 1,474 patients with AMI (71% men, 73 ± 14 years, 39% with STEMI) were consecutively included in a single-center registry of a tertiary hospital. Details regarding the registry design, methods, and quality control have been previously published^15^. AMI was defined according to the criteria set by international guidelines^16^. LVEF was measured throughout hospitalization at the discretion of the healthcare team. For this analysis, the worst LVEF of each patient during hospitalization was selected. Baseline clinical characteristics and in-hospital outcomes (length of stay and in-hospital mortality) were compared among the three groups of patients: those without HF at admission (Killip score = 1), those with HF with preserved LVEF at admission (Killip score > 1 and LVEF ≥ 50%), and those with systolic dysfunction at admission (Killip score > 1 and LVEF < 50%). The diagnosis of AMI and all decisions regarding the treatment administered were made by the responsible medical team based on the institution’s current guidelines and routine practices. Specific nursing staff was assigned to collect all the variables included in this registry. The Research Ethics Committee of Hospital Israelita Albert Einstein approved the present study.

### Statistical analyses

The numerical variables with normal distribution were expressed as mean ± standard deviation or as median and interquartile range when the distribution was not normal. Categorical variables were presented as absolute and relative frequencies. The comparison between numerical variables was performed using analysis of variance or the Kruskal–Wallis test, followed by the Bonferroni–Dunn multiple comparisons test, when required. The chi-squared test was used for categorical variables; Bonferroni multiple comparisons via generalized linear models with logit link function were used when the differences between the groups were significant. A logistic regression model adjusted for sex, age, troponin, diabetes mellitus, body mass index, type of AMI, and history of prior stroke/transient ischemic attack was used to test the association between HF and in-hospital mortality. A p-value < 0.05 was considered to be statistically significant. All statistical analyses were performed using STATA 11 Special Edition (Stata Corp LP, College Station, Texas, United States).

## Results

Among the 1,474 patients included in the study, 1,256 (85.2%) did not have HF (72% men, 67 ± 15 years), 78 (5.3%) had HF with preserved LVEF (59% men, 76 ± 14 years), and 140 (9.5%) had systolic HF (69% men, 76 ± 14 years). The baseline clinical characteristics of the three groups are shown in Table 1. It was observed that HF patients with preserved LVEF and those with systolic HF were older and had higher risk for thrombolysis in myocardial infarction (TIMI) than patients without HF. Compared with patients with systolic HF, HF patients with preserved LVEF had higher LVEF and often exhibited NSTEMI.

**Table 1:** Clinical characteristics of the three groups of patients

	**Without HF (n = 1,256)**	**HF with preserved LVEF (n = 78)**	**Systolic HF (n = 140)**	**p-value**
Male, n (%)	910 (72)	46(59)	96 (69)	0,028*
Age (±SD)	67 ± 15**,***	76 ± 14****	76 ± 14****	**< 0,001**
BMI (kg/m^2^) (±SD)	27 ± 4***	26 ± 5****	26 ± 5****	**0,015**
Diabetes, n (%)	370 (30)	30(39)	50 (36)	0,130
SAH,n(%)	700 (58)	52 (67)	86 (63)	0,157
Stroke/TIA, n (%)	48 (4)***	4 (5)	15 (11)****	**0,005**
Previous AMI, n (%)	195 (16)***	8 (11)	31 (23)****	**0,050**
LVEF (±SD)	0,54 ± 0,12**,***	0,59 ±0,07***,****	0,34 ±0,09**,****	**< 0,001**
TIMI risk (P25/P75)	2 (1/4)**,***	3 (2/6)****	4 (3/6,75)****	**< 0,001**
Troponin ng/mL (P25/P75)	3.460 (580/16.100)	3.160 (450/21.200)	3.300 (420/21.500)	0,940
NSTEMI, n (%)	790 (63)***	47 (60)***	60 (43)**,****	**< 0.001**

* It was not possible to identify the groups in which the differences occurred.

** Statistically significant differences compared with HF patients with preserved LVEF.

*** Statistically significant differences compared with systolic HF patients.

**** Statistically significant differences compared with patients without HF. HF: heart failure. LVEF: left ventricular ejection fraction. SD: standard deviation. BMI: body mass index. SAH: systemic arterial hypertension. TIA: transient ischemic attack. AMI: acute myocardial infarction. TIMI: thrombolysis in myocardial infarction. NSTEMI: acute myocardial infarction without ST-segment elevation.

Patients without HF, with HF with preserved LVEF, and with systolic HF presented mortality rates of 4.3%, 17.9%, and 27.1%, respectively (p < 0.001), and hospital stay (standard deviation) of 6 (5), 9 (14), and 10 (12.5) days, respectively (p < 0.001). Logistic regression revealed that HF with preserved LVEF [odds ratio (OR) = 2.91, 95% confidence interval (95% CI) 1.35–6.27, p = 0.006] and systolic dysfunction (OR = 5.38, 95% CI 3.10–9.32, p < 0.001) were notably and independently associated with in-hospital mortality (Table 2).

**Table 2:** Multivariate logistic regression

**Variable**	**OR**	**95% CI**		**p-value**
		**Lowest**	**Highest**	
HF with preserved LVEF	2,91	1,35	6,27	0,006
Systolic HF	5,38	3,10	9,32	< 0,001
Age (years)	1,02	1,01	1,03	0,003
BMI (kg/m^2^)	0,84	0,81	0,88	< 0,001
Female sex	1,44	0,87	2,41	0,160
Diabetes	0,88	0,53	1,45	0,615
Previous AMI	0,80	0,42	1,50	0,482
Previous stroke/TIA	2,02	0,91	4,48	0,085
Troponin	1,00	1,00	1,00	0,996
NSTEMI	0,66	0,40	1,07	0,092

OR: odds ratio. 95% CI: 95% confidence interval. HF: heart failure. LVEF: left ventricular ejection fraction. BMI: body mass index. AMI: acute myocardial infarction. TIA: transient ischemic attack. NSTEMI: acute myocardial infarction without ST-segment elevation.

## Discussion

One-third of patients with AMI who had HF at admission presented preserved LVEF (≥ 50%). Nevertheless, this subgroup had an extended hospital stay and an almost three-fold higher risk of in-hospital death than those without HF. Patients admitted with systolic HF exhibited even higher mortality rates, with a five-fold greater risk of in-hospital death when compared with patients without HF. 

Diastolic HF is a clinical syndrome characterized by the presence of signs and symptoms of HF, preserved LVEF, and abnormal diastolic function. The pathophysiology of diastolic HF comprises ventricular relaxation deficit and intraventricular pressure increase, with a consequent increase in pulmonary capillary wedge pressure^1^. In general, post-AMI HF is a result of complex and unbalanced structural, hemodynamic, and neurohumoral interactions^17^. Ischemia and myocardial necrosis promote systolic and diastolic contractile dysfunction because ventricular diastole is an active physiological process that consumes oxygen and glucose^18^. Even without extensive necrosis, a stunned or hibernating myocardium also presents contractile and relaxation dysfunction, although this may be transitory^19^.

Echocardiographic assessment of diastolic function and filling pressures requires careful data acquisition and proper interpretation by the operating technician. Decreases in the magnitude of the early to late diastolic filling ratio, increases in the deceleration time of early diastolic filling, or increases in the isovolumetric relaxation time indicate worsened ventricular relaxation^19^. These echocardiographic parameters can aid in diagnosis and assessment of the severity of diastolic dysfunction. A 2007 European consensus suggested that, in addition to the clinical characteristics of HF and LVEF, echocardiographic parameters such as ventricular filling time, diastolic volume, and ventricular mass should be included among the diagnostic criteria for diastolic HF^20^. Data on these parameters were not available in our registry; nevertheless, as in the present study, the majority of clinical studies on post-AMI diastolic dysfunction have used only clinical HF associated with preserved LVEF to establish the diagnosis^12,21^.

In patients with acute coronary syndromes (ACS), the presence of HF is an important marker for risk of death. Stege et al. evaluated the characteristics and prognosis of post-ACS HF based on the GRACE registry^13^. They observed a 2.2-fold higher risk of death for patients with HF than those without HF. Notably, the GRACE registry did not differentiate according to patients the type of HF (systolic or diastolic) but classified them only based on the Killip score at admission. In addition, patients with Killip class IV AMI were excluded from the analysis, which may justify the lower mortality in these patients compared with the results of our or other registries^14,22^.

Conversely, the data available on diastolic dysfunction in patients with ACS are highly limited. Patients with ACS and HF often have preserved LVEF; nevertheless, most clinical studies have only analyzed the outcomes of patients with systolic HF. Recently, an epidemiological study demonstrated that, despite the prevalence of post-AMI systolic HF declining over the past two decades, prevalence for HF with preserved LVEF has remained stable, reaching a rate comparable with systolic HF^23^. In general, patients with HF with preserved LVEF are majorly women, the elderly, hypertensive individuals, and those with lower prevalence of diabetes mellitus compared with patients with post-ACS systolic HF^12^. In the present study, compared with patients without HF, patients with systolic HF or HF with preserved LVEF were older and had a higher risk of AMI, as assessed by the TIMI score. Compared with patients with systolic HF, HF patients with preserved LVEF had higher LVEF and often exhibited NSTEMI.

In relation to clinical outcomes, some studies have shown that patients with post-AMI HF and preserved LVEF had higher risk of mortality compared with patients without HF, despite not exhibiting systolic dysfunction. Bennett et al. found results similar to the present study in the CRUSADE registry, specifically in patients with NSTEMI^12^. In that registry, over half the patients with post‑AMI HF had preserved LVEF. However, the cut-off point used to determine preserved LVEF was 40%. Therefore, patients with mild systolic ventricular dysfunction were considered to have diastolic HF, which may have worsened the prognosis of this subset of patients. Nevertheless, in the CRUSADE registry, mortality in patients with HF with preserved LVEF was lower than that in patients with systolic dysfunction^12^. Similarly to the present study, this rate was more than twice the rate in patients without HF. In the same registry, this behavior was also observed in the short- and long-term sub-analysis in patients aged over 65 years^24^. Notably, the CRUSADE registry did not include patients with STEMI, who represented 40% of HF patients with preserved LVEF in the study.

Subsequently, Kim et al.^22^ assessed predictors of death including NT-proBNP in 555 patients with AMI and preserved LVEF. Age and NT-proBNP were independent predictors of cardiovascular mortality and rehospitalization for HF. Recently, in a large registry (ACTION) analyzed by Shah et al.,^21^ 3.8% of patients with AMI admitted without HF developed HF during hospitalization. In this subgroup, 35% of patients exhibited NSTEMI and 22% of those exhibiting STEMI developed HF with LVEF ≥ 50%. Despite mortality in patients with post-AMI HF being approximately five times greater than in those without HF, they did not observe differences in mortality between patients with systolic HF and those with HF with preserved LVEF. However, the study suggested that preserved LVEF and absence of HF at admission did not guarantee that patients with AMI were free from the risk of developing HF during hospitalization.

The present study had several limitations because this was a retrospective, observational, single-center study with a relatively small population sample. Data on the patients’ Killip score throughout hospitalization was not available but only that upon admission was available; therefore, this study did not include cases of HF that developed during hospitalization. Echocardiographic measurements related to diastolic function other than the LVEF score were also not available in the present registry. Finally, complete data on the medical and interventional treatment of the patients were not available, and as a result, statistical adjustments related to therapeutic aspects were not possible.

Thus, although post-AMI HF with preserved LVEF is moderately prevalent and presents important prognostic implications, few studies have specifically evaluated the clinical outcomes and therapeutic needs of this subgroup of patients. Despite its limitations, the objective of this study was to describe the clinical features, prevalence, and prognosis of patients with systolic HF or HF with preserved LVEF following AMI.

## Conclusion

One-third of patients with AMI with HF at admission presented preserved LVEF. Although outcomes for this subgroup were more favorable than those for the patients with systolic HF, the former had longer hospital stays and a three-fold higher risk of death than the patients without HF. Therefore, HF patients with preserved LVEF after AMI are a subgroup encountering a short-term risk and require special attention and monitoring during hospitalization.
